# Genetic Analysis of *Candida auris* Implicates Hsp90 in Morphogenesis and Azole Tolerance and Cdr1 in Azole Resistance

**DOI:** 10.1128/mBio.02529-18

**Published:** 2019-01-29

**Authors:** Sang Hu Kim, Kali R. Iyer, Lakhansing Pardeshi, José F. Muñoz, Nicole Robbins, Christina A. Cuomo, Koon Ho Wong, Leah E. Cowen

**Affiliations:** a Department of Molecular Genetics, University of Toronto, Toronto, Ontario, Canada; b Genomics and Bioinformatics Core, Faculty of Health Sciences, University of Macau, Macau, China; c Broad Institute of Harvard and Massachusetts Institute of Technology, Cambridge, Massachusetts, USA; d Faculty of Health Sciences, University of Macau, Macau, China; e Institute of Translational Medicine, University of Macau, Macau, China; University of British Columbia

**Keywords:** antifungal drug resistance, *Candida albicans*, *Candida auris*, Cdr1, developmental program, emerging pathogen, fungal morphogenesis, fungal pathogen, Hsp90, transcriptional program

## Abstract

Candida auris is an emerging fungal pathogen and a serious global health threat as the majority of clinical isolates display elevated resistance to currently available antifungal drugs. Despite the increased prevalence of C. auris infections, the mechanisms governing drug resistance remain largely elusive. In diverse fungi, the evolution of drug resistance is enabled by the essential molecular chaperone Hsp90, which stabilizes key regulators of cellular responses to drug-induced stress. Hsp90 also orchestrates temperature-dependent morphogenesis in Candida albicans, a key virulence trait. However, the role of Hsp90 in the pathobiology of C. auris remains unknown. In order to study regulatory functions of Hsp90 in C. auris, we placed *HSP90* under the control of a doxycycline-repressible promoter to enable transcriptional repression. We found that Hsp90 is essential for growth in C. auris and that it enables tolerance of clinical isolates with respect to the azoles, which inhibit biosynthesis of the membrane sterol ergosterol. High-level azole resistance was independent of Hsp90 but dependent on the ABC transporter *CDR1*, deletion of which resulted in abrogated resistance. Strikingly, we discovered that C. auris undergoes a morphogenetic transition from yeast to filamentous growth in response to *HSP90* depletion or cell cycle arrest but not in response to other cues that induce C. albicans filamentation. Finally, we observed that this developmental transition is associated with global transcriptional changes, including the induction of cell wall-related genes. Overall, this report provides a novel insight into mechanisms of drug tolerance and resistance in C. auris and describes a developmental transition in response to perturbation of a core regulator of protein homeostasis.

## Abstract (precis)

Fungal pathogens pose a serious threat to public health. Candida auris is an emerging fungal pathogen that is often resistant to commonly used antifungal drugs. However, the mechanisms governing drug resistance and virulence in this organism remain largely unexplored. In this study, we adapted a conditional expression system to modulate the transcription of an essential gene, *HSP90*, which regulates antifungal resistance and virulence in diverse fungal pathogens. We showed that Hsp90 is essential for growth in C. auris and is important for tolerance of the clinically important azole antifungals, which block ergosterol biosynthesis. Further, we established that the Cdr1 efflux transporter regulates azole resistance. Finally, we discovered that C. auris transitions from yeast to filamentous growth in response to Hsp90 inhibition, accompanied by global transcriptional remodeling. Overall, this work provides a novel insight into mechanisms regulating azole resistance in C. auris and uncovers a distinct developmental program regulated by Hsp90.

## INTRODUCTION

Antimicrobial resistance is becoming an increasing global public health burden now that multidrug resistance in pathogens has transitioned from a rare curiosity to a frequent occurrence, eroding our ability to effectively control infections with antimicrobials. The emergence of drug resistance in fungal pathogens is of particular concern given the increasing incidence of mycotic infections, with approximately 1.5 million people succumbing to invasive fungal infections per year despite therapeutic intervention (1). Further, there remains a paucity of safe and effective antimicrobials, particularly for antifungals, for which only three major drug classes have been approved to treat systemic infections. Thus, there exists a dire need for new strategies to prevent the evolution of drug resistance and enhance the efficacy of antifungal drugs.

Although they are commensals of the human microbiota, *Candida* species are capable of causing life-threatening systemic disease in immunocompromised individuals. *Candida* species account for 88% of all hospital-acquired fungal infections in the United States, with Candida albicans being the primary cause of candidiasis exhibiting mortality rates of ∼40%, even with current treatments (2, 3). The recent emergence of Candida auris has caused significant concern given its worldwide distribution and high reported incidence of antifungal resistance (4, 5). Specifically, studies have estimated that as much as 93% of clinical isolates exhibit increased resistance to fluconazole, an azole commonly administered for treating systemic *Candida* infections (6). Most alarmingly, some isolates have been reported to show elevated resistance to all three major antifungal classes, leaving no treatment options for such infections (4). Despite the high incidence of azole resistance reported for C. auris isolates, little is known about the mechanisms involved. Consequently, this pathogen represents a great concern for public health agencies given the possibility that biological and epidemiological factors could trigger an even more extensive worldwide epidemic of C. auris infections.

The most widely deployed class of antifungal is the azoles, which inhibit fungal growth by targeting lanosterol 14-α-demethylase (Erg11), a key component of the pathway for biosynthesis of the membrane sterol ergosterol (7). Azole resistance mechanisms have been studied most extensively in C. albicans and include alteration or overexpression of the target, activation or increased expression of azole efflux mediators, and induction of cellular stress responses (7). Specifically, in C. albicans, the molecular chaperone Hsp90 promotes the evolution of drug resistance by stabilizing regulators of cellular responses to drug-induced stress (8). Inhibition of Hsp90 blocks calcineurin-dependent stress responses and cell wall integrity signaling, thereby reducing antifungal tolerance of clinical isolates and transforming azole activity from fungistatic to fungicidal (8–11). Moreover, the impact of Hsp90 on drug resistance has been conserved in a number of other fungal pathogens, including Candida glabrata and Aspergillus fumigatus (12–14). In C. auris, studies have suggested that point mutations in *ERG11* contribute to azole resistance of clinical isolates (15). Further, the identification of many putative transporter genes in the C. auris genome suggests that drug efflux may also be an important determinant of azole resistance (16–18). Despite these initial reports, genetic control of azole resistance has yet to be explored in C. auris.

In addition to its role in enabling the emergence and maintenance of azole resistance, Hsp90 governs temperature-dependent morphogenesis in C. albicans (19). The ability of C. albicans to transition between yeast and filamentous forms is a key virulence trait that is triggered by a wide variety of environmental cues (20). Hsp90 represses filamentous growth in C. albicans such that compromising Hsp90 function induces the yeast-to-filament transition by relieving repression on Ras1-protein kinase A (PKA) signaling (19). Hsp90 also controls morphogenesis via the Pcl1 cyclin, the Pho85 and Cdc28 cyclin-dependent kinases, and the Hms1 transcription factor, as well as by additional mechanisms that remain enigmatic (21, 22). However, exposure to various cues that induce C. albicans filamentation does not induce morphogenesis in C. auris, suggesting divergence in the circuitry governing the yeast-to-filament transition between the two *Candida* species (23).

In this study, we sought to explore the role of the Hsp90 molecular chaperone in regulating drug resistance and virulence in C. auris. Genetic depletion or pharmacological inhibition of Hsp90 had no effect on fluconazole resistance; however, it was important for the azole tolerance of some clinical isolates, potentially enabling the evolution of drug resistance in otherwise fluconazole-sensitive strains. Despite the presence of several putative ABC transporter genes in the C. auris genome, deletion of *CDR1* was sufficient to confer an 8-fold increase in the sensitivity of C. auris to fluconazole, indicating that this specific efflux transporter plays a pivotal role in azole resistance. Finally, we discovered that genetic depletion or pharmacological inhibition of Hsp90 induced filamentous growth in C. auris. Global transcriptional analysis highlighted that compromise of Hsp90 function was accompanied by transcriptional upregulation of predicted cell surface genes for which the C. albicans orthologs have functional annotations associated with filamentous growth. Thus, this study implicated Cdr1-mediated efflux as a key determinant of azole resistance in C. auris and provided the first evidence that perturbation of a core regulator of protein homeostasis controls the morphogenesis of this emerging pathogen.

## RESULTS

### *HSP90* is essential in C. auris.

**FIG 1:**
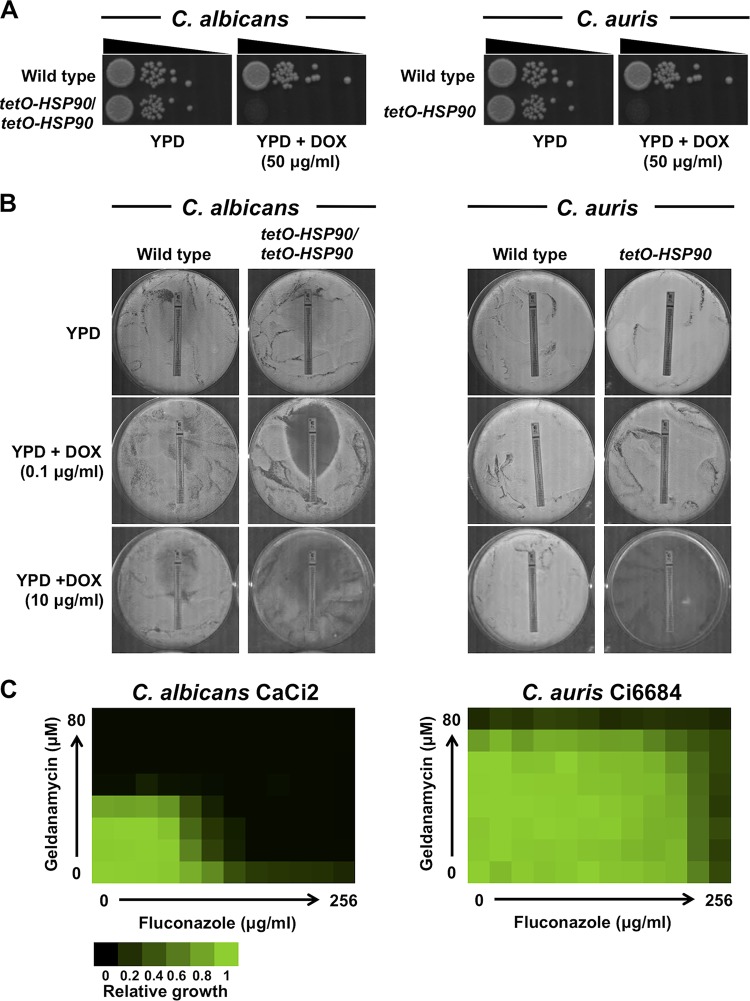
Perturbation of *HSP90* results in a loss of viability of C. auris but does not affect azole resistance. (A) Spotting of C. auris wild-type and *tetO-HSP90* strains on YPD or YPD agar plus doxycycline (DOX) plates (right panel). C. albicans wild-type and *tetO-HSP90*/*tetO-HSP90* strains were included for comparison (left panel). A high concentration (50 μg/ml) of DOX was used to ensure strong repression of *HSP90* expression. Overnight cultures were diluted 1,000-fold, at which point 5 μl was spotted in 100-fold dilutions. Plates were incubated at 30°C for 48 h. (B) Fluconazole Etest strip in the presence and absence of DOX. A total of 1 × 10^6^ cells of wild-type and *tetO-*repressible *HSP90* strains were plated on YPD agar plates with fluconazole Etest strips in the absence and presence of DOX (0.1 μg/ml or 10 μg/ml). The plates were incubated at 30°C for 48 h. (C) Checkerboard assays with geldanamycin and fluconazole. C. albicans clinical isolate CaCi2 and C. auris isolate Ci6684 were inoculated with a 2-fold gradient of geldanamycin in combination with a 2-fold gradient of fluconazole. Cultures were incubated at 30°C for 48 h. Heat maps represent relative growth levels determined from averages of results of technical replicates normalized to the data from a no-drug well.

A powerful approach to explore essential gene function is by engineering conditional expression strains in which the native promoter of a gene is replaced with a regulatable promoter to enable transcriptional induction or repression. To investigate the impact of Hsp90 on C. auris drug resistance and virulence, we constructed a *tetO-HSP90* strain by targeting the *HSP90* promoter with Cas9/single guide RNA (sgRNA) and replacing the native promoter with the tetracycline-repressible *tetO* promoter system in genome-sequenced, fluconazole-resistant C. auris strain Ci6684 (see Fig. S1A in the supplemental material) (16). This was achieved by adapting a C. albicans clustered regularly interspaced short palindromic repeat (CRISPR)/Cas9-mediated promoter replacement system (24). To confirm proper integration of the *tetO* repair cassette upstream of the *HSP90* locus, PCR genotyping was employed (Fig. S1A). Once transformants were successfully genotyped, the conditional expression of *HSP90* was verified. We observed that the *tetO-HSP90* strain expressed *HSP90* at a higher level than the wild-type control in the absence of the tetracycline analog doxycycline (Fig. S1B). Upon the addition of doxycycline, *HSP90* levels were reduced to less than 40% of that seen with the wild-type control (Fig. S1B). Next, in order to test the essentiality of *HSP90* in C. auris, the *tetO-HSP90* strain was serially diluted and spotted onto an agar plate containing a high concentration of doxycycline. Given that *HSP90* is essential in C. albicans (19, 25), a C. albicans *tetO-HSP90*/*tetO-HSP90* strain was included as a control. As observed with the C. albicans *HSP90* conditional expression strain, doxycycline-mediated transcriptional repression of C. auris *HSP90* resulted in a lack of viable colonies recovered via spotting (Fig. 1A). Thus, Hsp90 is essential in C. auris, and the *tetO* promoter system offers a powerful approach for studying essential gene function in this emerging pathogen.

10.1128/mBio.02529-18.1 FIG S1 Validating an *HSP90* conditional expression system in C. auris. (A) Schematic representation of wild-type and *tetO-HSP90* genotypes (left panel) and results of PCR genotyping (right panel). Oligonucleotide pairs used for verification of upstream integration (u), downstream integration (d), and the absence of the native promoter (n) are listed above the locus. *HygB*, hygromycin resistance marker; *TA*, tetracycline-repressible activator; *tetO*, activator binding sequence. (B) *HSP90* transcript levels as measured by qRT-PCR of Ci6684 wild-type and *tetO-HSP90* strains in the presence and absence of doxycycline (DOX). *HSP90* transcript levels were normalized against *ACT1* and *GPD1* transcript levels and then compared to those seen under the wild-type (WT) YPD-only condition. *, *P* < 0.05 (one-way analysis of variance [ANOVA]). Download FIG S1, TIF file, 0.7 MB. Copyright © 2019 Kim et al. 2019 Kim et al. This content is distributed under the terms of the Creative Commons Attribution 4.0 International license.

### Hsp90 is an important mediator of tolerance in C. auris.

Given that Hsp90 is crucial for enabling cellular responses to azole-induced cell membrane stress in diverse fungi, including C. albicans, C. glabrata, and A. fumigatus (8, 10, 14), we tested whether Hsp90 had an impact on azole resistance in C. auris. To do so, the C. auris *tetO-HSP90* strain was plated on rich medium without or with doxycycline at various concentrations and growth was monitored in the presence of a fluconazole Etest strip, a commercial antifungal strip with a defined gradient of fluconazole, to determine the minimum inhibitory concentration (MIC). The C. albicans *tetO-HSP90*/*tetO-HSP90* strain was included as a control (12). In the absence of doxycycline, both C. auris and C. albicans were capable of growing at concentrations of fluconazole up to the highest tested (Fig. 1B). As expected, incubation of the C. albicans *tetO-HSP90*/*tetO-HSP90* strain with a doxycycline concentration that did not affect growth on its own (0.1 μg/ml) caused hypersensitivity to fluconazole. However, the same doxycycline concentration had no effect on growth of the C. auris *tetO-HSP90* strain in the presence of fluconazole (Fig. 1B). A further increase in the concentration of doxycycline to 10 μg/ml severely affected growth on its own in both C. albicans and C. auris due to *HSP90* essentiality (Fig. 1B). As a complementary approach, the impact of Hsp90 on azole resistance was evaluated in liquid culture using a standard checkerboard assay. C. auris strain Ci6684 was inoculated in rich medium containing a 2-fold dilution series of concentrations of fluconazole or geldanamycin, which inhibits Hsp90 chaperone function (26). As a control, a C. albicans clinical isolate (strain CaCi2) recovered from an HIV-infected patient at an early stage of treatment with fluconazole was also employed, as inhibition of Hsp90 is known to enhance the azole susceptibility of this strain (8, 27). Although geldanamycin potentiated fluconazole activity against C. albicans, the combination had no effect on azole susceptibility in C. auris (Fig. 1C).

**FIG 2:**
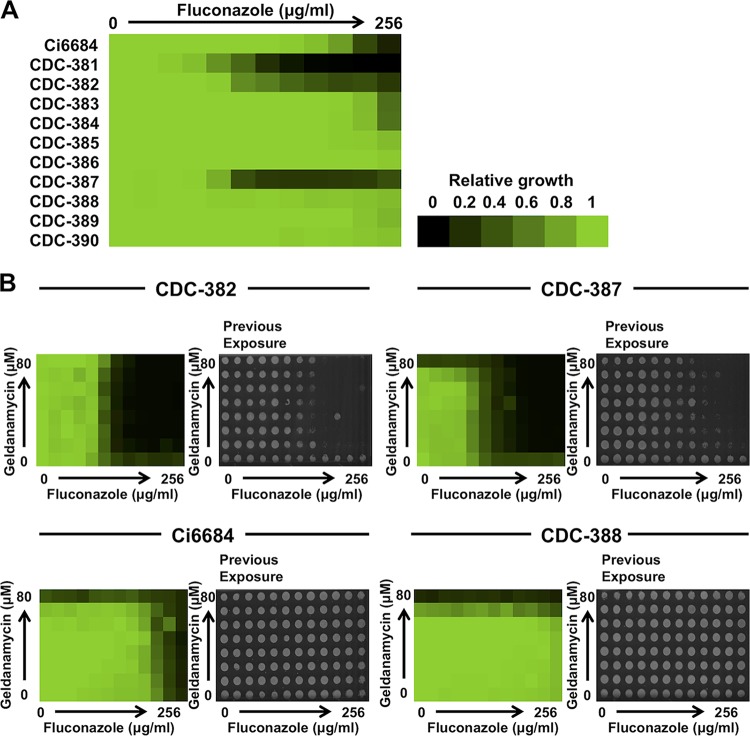
Hsp90 mediates tolerance of fluconazole in C. auris. (A) MIC assay for fluconazole in a panel of C. auris clinical isolates. Data were analyzed as described for Fig. 1C. (B) Checkerboard assays with geldanamycin and fluconazole. C. auris strains Ci6684, CDC-382, CDC-387, and CDC-388 were inoculated with a 2-fold gradient of geldanamycin in combination with a 2-fold gradient of fluconazole. Data were analyzed as described for Fig. 1C. After measurement of the growth, cultures were spotted onto drug-free YPD agar plates and allowed to grow at 30°C for 24 h to check for cidality.

Next, we expanded our analysis of the impact of Hsp90 on azole resistance to a broader panel of C. auris clinical isolates provided by the Centers for Disease Control and Prevention (CDC) (Fig. 2A) (6). Three clinical isolates, CDC-381, CDC-382, and CDC-387, were more susceptible to fluconazole than the other strains. Of these, CDC-381 and CDC-382 showed a tolerance phenotype with persistent growth beyond the drug MIC (Fig. 2A). In order to assess the role of Hsp90 in tolerance of fluconazole in these isolates, we performed checkerboard assays with the combination of fluconazole and geldanamycin. For strains CDC-382 and CDC-387, geldanamycin abrogated tolerance of fluconazole at concentrations above their fluconazole MICs (Fig. 2B), a result which was also recapitulated in an Etest strip assay (Fig. S2). Furthermore, the combination transformed the fungistatic activity of fluconazole to fungicidal, as observed upon attempted culturing on drug-free medium following drug exposure (Fig. 2B). Notably, geldanamycin did not affect the high-level fluconazole resistance phenotypes of strain Ci6684 or strain CDC-388 or the fungistatic activity of fluconazole against these strains (Fig. 2B). This is akin to what has been observed in C. albicans in the context of clinical isolates that overexpress efflux pumps and harbor mutations in the *ERG11* drug target gene and which often exhibit azole resistance phenotypes that are independent of Hsp90 (8, 28). Overall, our work implicates Hsp90 as an important mediator of fluconazole tolerance in C. auris.

10.1128/mBio.02529-18.2 FIG S2 Pharmacological inhibition of Hsp90 reduces C. auris growth in fluconazole-susceptible strains. Data represent results of a fluconazole Etest strip assay in the presence and absence of geldanamycin. A total of 1 × 10^6^ cells of C. auris strains Ci6684, CDC-382, and CDC-387 were plated on YPD agar plates with fluconazole Etest strips in the absence and presence of geldanamycin (40 μM). The plates were incubated at 30°C for 48 h and then imaged. Download FIG S2, TIF file, 1.8 MB. Copyright © 2019 Kim et al. 2019 Kim et al. This content is distributed under the terms of the Creative Commons Attribution 4.0 International license.

### Hsp90-independent fluconazole resistance is mediated in part by Cdr1.

**FIG 3:**
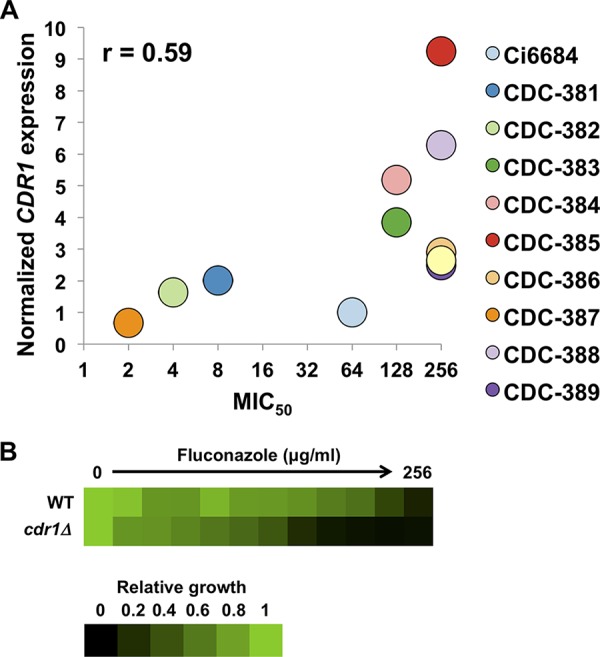
Fluconazole resistance is mediated by drug efflux in C. auris. (A) Plot of relative *CDR1* expression versus MIC_50_ in the panel of C. auris isolates. *CDR1* transcript levels were normalized against *ACT1* and *GPD1* transcript levels. MIC_50_ values were derived from the MIC assay results presented in Fig. 2. The Pearson correlation coefficient (r) was calculated for the two values. (B) MIC assay for fluconazole. Ci6684 (wild type [WT]) and *cdr1Δ* strains were inoculated with a 2-fold gradient of fluconazole. The plates were incubated at 30°C for 48 h. Data were analyzed as described for Fig. 1C.

Given that Hsp90 had minimal impact on the high level of azole resistance displayed by several C. auris strains, we explored another mechanism by which azole resistance might be regulated in this emerging pathogen. We focused on drug efflux, as this is a major contributor to azole resistance in diverse fungi (7). In order to determine if drug efflux is an important mechanism of azole resistance in C. auris, we measured transcript levels of the *CDR1* ABC transporter gene in the CDC panel of clinical isolates. When we compared the MIC_50_ values of fluconazole for each isolate to their *CDR1* expression level, we observed a positive correlation (*r* [Pearson correlation coefficient] = 0.59) (Fig. 3A), suggesting that increased expression of *CDR1* may contribute to elevated fluconazole resistance. To test this genetically, we deleted the *CDR1* open reading frame in the Ci6684 background and observed an 8-fold decrease in fluconazole MIC (Fig. 3B), suggesting that Cdr1 is a major contributor to azole resistance in C. auris.

### Hsp90 represses filamentous growth in C. auris.

**FIG 4:**
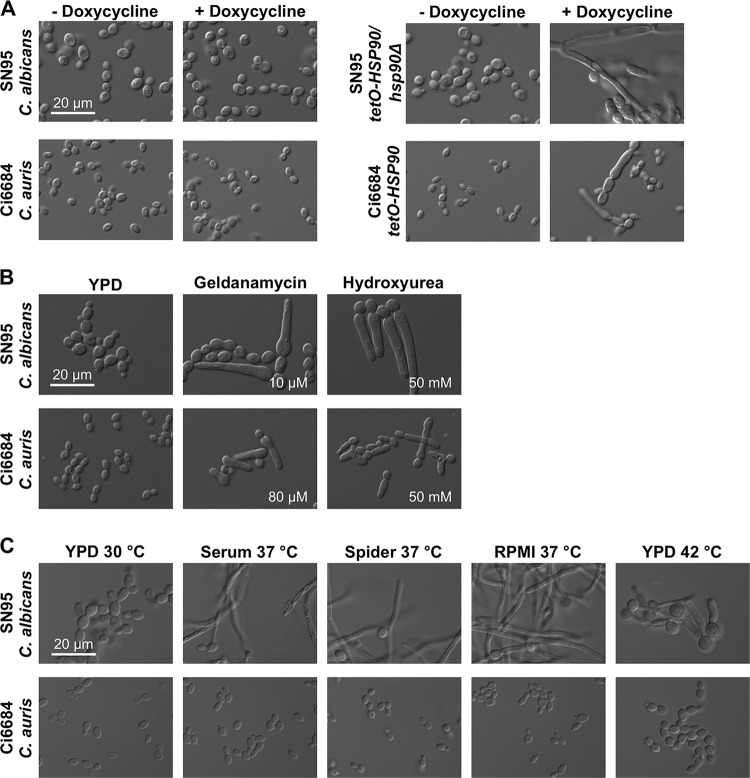
C. auris undergoes filamentous growth under conditions of compromised Hsp90 function. (A) Overnight cultures of C. albicans (strain SN95) and C. auris (strain Ci6684) or of the respective strains with *HSP90* under the control of a *tetO*-repressible promoter were subcultured in YPD without or with doxycycline. Images were captured after 6 h postsubculture. (B) Overnight cultures of wild-type C. albicans and C. auris strains were subcultured in YPD without or with geldanamycin (10 μM for C. albicans and 80 μM for C. auris) or hydroxyurea (50 mM). Images were captured after 6 h of drug treatment. (C) Overnight cultures of C. albicans and C. auris were subcultured in canonical C. albicans filament-inducing cues. These include 37°C plus 10% serum (Serum), 37°C plus Spider medium (Spider), 37°C RPMI medium, and 42°C. Microscopy images were acquired after 6 h of incubation. The scale bars represent 20 μm in all panels.

In various fungal pathogens, Hsp90 regulates not only drug resistance but also morphogenesis (19, 29, 30). During characterization of the C. auris *tetO-HSP90* strain, we discovered that depletion of *HSP90* resulted in polarized growth, similar to what was observed in C. albicans (Fig. 4A). This was surprising, as filamentous growth had previously been described in C. auris only in response to a high salt concentration at elevated temperatures (31). Consistent with genetic depletion of *HSP90*, high concentrations of the Hsp90 inhibitor geldanamycin also induced filamentous growth, implicating Hsp90 as a key regulator of morphogenesis in this pathogen (Fig. 4B). C. auris did not filament in response to canonical filament-inducing cues for C. albicans, such as serum, Spider medium, RPMI medium, or elevated temperature (Fig. 4C). This is akin to other reports that C. auris does not filament in response to Lee’s medium and GlcNAc medium (31). However, high doses of the cell cycle arresting agent hydroxyurea induced polarized growth in C. auris, similar to what was observed with C. albicans (Fig. 4B). Thus, C. auris is capable of undergoing a distinct morphogenetic transition in response to cell cycle arrest or Hsp90 inhibition.

**FIG 5:**
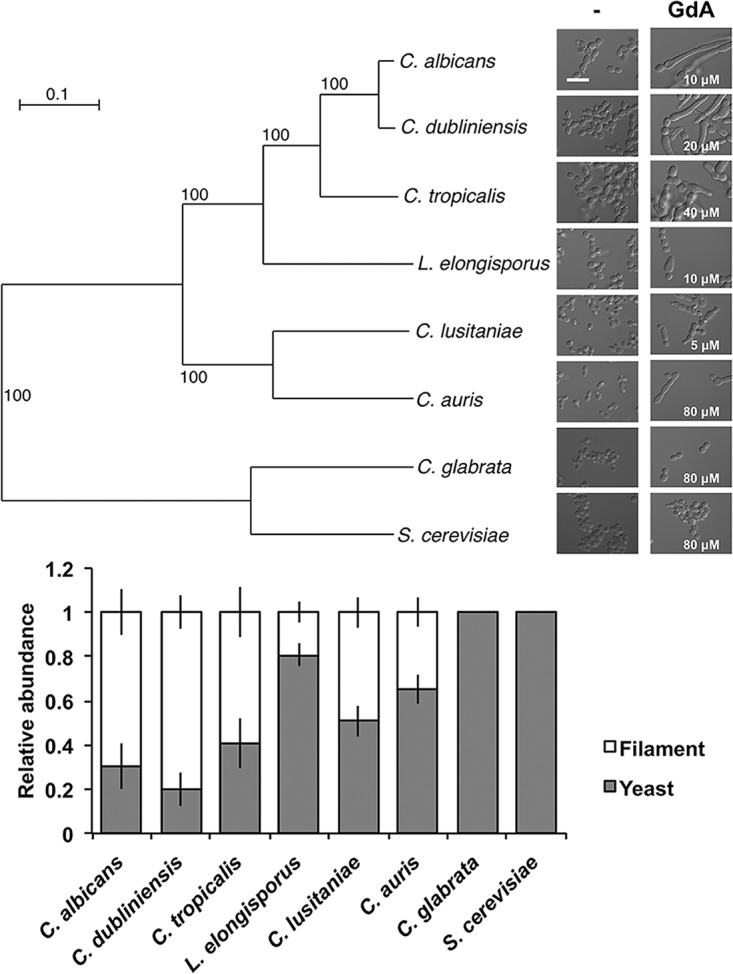
CUG clade species undergo filamentous growth under conditions of compromised Hsp90 function. Overnight cultures of C. albicans, C. dubliniensis, C. tropicalis, L. elongisporus, C. lusitaniae, C. auris, S. cerevisiae, and C. glabrata were subcultured in YPD without or with geldanamycin (GdA). (Top left) A phylogenetic tree of the yeast species was constructed using 1,570 single-copy protein-coding genes, maximum likelihood inference based on 1,000 replicates, and RAxML v7.7.8. Scale bar, mean number of nucleotide substitutions per site. (Top right) Microscopy images were acquired after 6 h of incubation. Scale bar, 20 μm. The concentration of geldanamycin used for each species is indicated on the micrograph. (Bottom) Proportions of yeast to filament were quantified.

To test whether filamentous growth in response to Hsp90 inhibition is a distinct trait in C. albicans and C. auris or a general phenotype in other *Saccharomycetales* yeasts, we monitored cellular morphology in Saccharomyces cerevisiae, Candida glabrata, Candida albicans, Candida dubliniensis, Candida tropicalis, Lodderomyces elongisporus, Candida lusitaniae, and C. auris in response to the presence of the Hsp90 inhibitor geldanamycin at a concentration close to the MIC (Fig. S3). Interestingly, all species classified within the “CUG” clade showed filamentous growth in response to geldanamycin to various degrees, while the non-CUG clade species remained exclusively in yeast form (Fig. 5). Therefore, filamentation in response to Hsp90 inhibition appears to be a trait conserved within the CUG clade.

10.1128/mBio.02529-18.3 FIG S3 MIC of geldanamycin for a panel of yeast species. Data represent results of a geldanamycin MIC assay of a panel of C. auris clinical isolates. Cells were inoculated with a two-fold gradient of geldanamycin. The plates were incubated at 30°C for 48 h, and then OD_600_ was measured. The heat map was plotted from averages of data from technical replicates normalized to the data from a no-drug well. Download FIG S3, TIF file, 0.1 MB. Copyright © 2019 Kim et al. 2019 Kim et al. This content is distributed under the terms of the Creative Commons Attribution 4.0 International license.

### C. auris filamentation is associated with transcriptional induction of putative cell surface genes.

The transcriptional remodeling that occurs during morphogenesis has been extensively studied in C. albicans (32, 33); however, such a response has never been monitored in C. auris. To characterize the C. auris filamentation program under conditions of Hsp90 perturbation, we examined the transcriptional changes upon genetic depletion and pharmacological inhibition of Hsp90 by RNA sequencing (RNA-Seq). The transcriptome of C. auris strain Ci6684 was assessed in the absence or presence of the Hsp90 inhibitor geldanamycin, while the transcriptome of the C. auris *tetO-HSP90* strain and its parent strain were assessed in the absence or presence of doxycycline. The corresponding experiments were also performed in C. albicans to compare the gene expression changes between the two organisms. The concentrations of geldanamycin and doxycycline used were sufficient to induce polarized growth (Fig. S4). The sequences identified through RNA-Seq were reference assembled to the B8441 reference genome, and C. auris transcripts were annotated based on a published putative transcript list (6, 16, 18, 34). Transcripts were assigned as representing a differentially expressed gene (DEG) if the fold change value for the drug-treated culture compared to the control culture was greater than ±1.5 and the false-discovery-rate (*q*) value was lower than 0.05. To control for any effects of doxycycline independently of the *tetO* promoter, transcriptomic changes in response to doxycycline were also measured in both species.

10.1128/mBio.02529-18.4 FIG S4 Confirmation of filamentous growth in cells used for RNA sequencing. Overnight cultures of wild-type C. albicans and C. auris and the respective strains with *HSP90* under the control of a *tetO*-repressible promoter were inoculated into 10 ml YPD, YPD agar plus doxycycline (0.5 μg/ml), or YPD agar plus geldanamycin (10 μM for C. albicans, 80 μM for C. auris) to the final OD_600_ of 0.1 and incubated at 30°C with shaking for 24 h. They were then subcultured to 25 ml of YPD, YPD agar plus doxycycline (5 μg/ml), or YPD agar plus geldanamycin (10 μM for C. albicans, 80 μM for C. auris) to the final OD_600_ of 0.1 and incubated at 30°C with shaking for 4 h. Scale bar, 20 μm. Download FIG S4, TIF file, 1.2 MB. Copyright © 2019 Kim et al. 2019 Kim et al. This content is distributed under the terms of the Creative Commons Attribution 4.0 International license.

**FIG 6:**
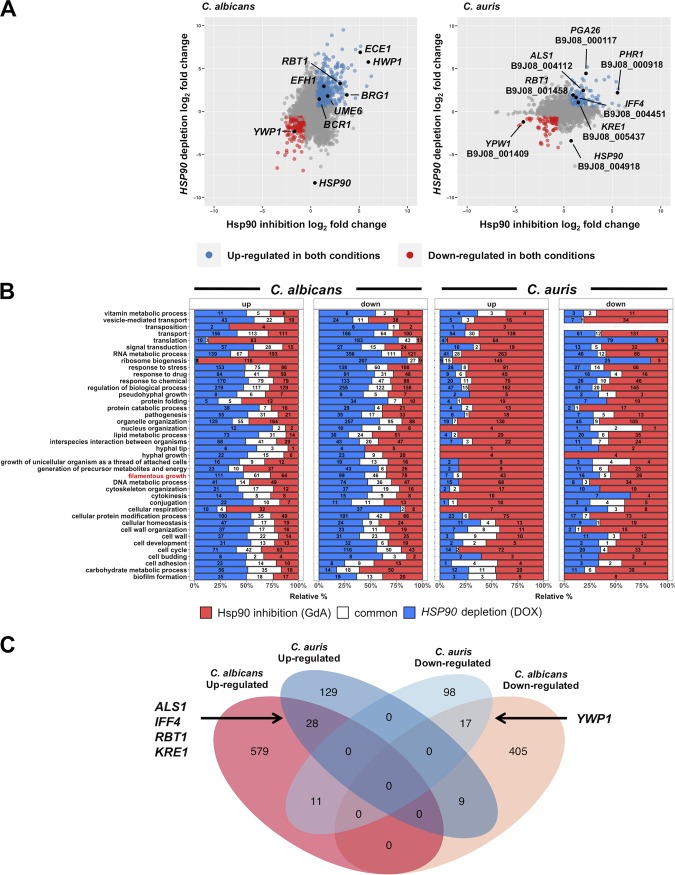
Global transcriptional response to Hsp90 perturbation in C. albicans and C. auris. (A) Scatter plot of log_2_ fold change values upon Hsp90 inhibition (*x* axis) and *HSP90* depletion (*y* axis) in C. albicans (left) and C. auris (right). Transcripts that were differentially regulated upon Hsp90 perturbation (log_2_ fold change of ±0.58 and *q* value of <0.05) are colored in blue (upregulated) or red (downregulated). Transcripts of interest are labeled in black. (B) GO slim mapping of differentially regulated transcripts in C. albicans (left) and C. auris (right). Transcripts differentially regulated under conditions of Hsp90 inhibition are colored in red, under conditions of *HSP90* depletion in blue, and under both conditions in white. Data corresponding to the GO slim term “filamentous growth” is highlighted in red. (C) Venn diagram of genes differentially expressed under conditions of Hsp90 inhibition and *HSP90* depletion in C. albicans and C. auris.

We identified global transcriptional changes in response to Hsp90 compromise in both species. In total, 2,501 and 2,111 DEGs were identified upon inhibition of Hsp90 with geldanamycin in C. albicans and C. auris, respectively (Fig. 6A; see also Table S1 in the supplemental material). We performed gene ontology (GO) term enrichment analysis of the DEGs in C. albicans and found that inhibition of Hsp90 was associated with upregulation of transcripts involved in mitochondrial respiration and ribosomal processes and with downregulation of transcripts involved in metabolic and biosynthetic processes (Fig. S5A and Table S2). In response to doxycycline-mediated transcriptional repression of *HSP90*, 3,331 and 1,014 DEGs were identified in C. albicans and C. auris, respectively (Fig. 6A; see also Table S1). As expected, *HSP90* was one of the most extensively downregulated transcripts in the depletion data set for both species (Table S1).

10.1128/mBio.02529-18.5 FIG S5 GO term enrichment of differentially regulated transcripts in response to Hsp90 perturbation in C. albicans. Data represent results of GO term enrichment of C. albicans transcripts generated by ClusterProfiler under the following conditions: (A) Hsp90 inhibition by geldanamycin and (B) doxycycline-mediated transcriptional repression of *HSP90* in the *tetO-HSP90*/*hsp90Δ* strain. The *y*-axis data show enriched GO terms, and the *x*-axis data show gene ratios. Adjusted *P* value (p.adjust) data are represented by the heat map, and the number of transcripts in each GO term (count) is represented by the size of each of the dots. Download FIG S5, TIF file, 2.3 MB. Copyright © 2019 Kim et al. 2019 Kim et al. This content is distributed under the terms of the Creative Commons Attribution 4.0 International license.

10.1128/mBio.02529-18.7 TABLE S1 Transcriptional profile of C. albicans and C. auris in response to Hsp90 inhibition and *HSP90* depletion by RNA-Seq. Download Table S1, XLSX file, 7.4 MB. Copyright © 2019 Kim et al. 2019 Kim et al. This content is distributed under the terms of the Creative Commons Attribution 4.0 International license.

10.1128/mBio.02529-18.8 TABLE S2 GO term enrichment analysis of differentially expressed genes in response to Hsp90 inhibition and *HSP90* depletion in C. albicans and C. auris. Download Table S2, XLSX file, 0.1 MB. Copyright © 2019 Kim et al. 2019 Kim et al. This content is distributed under the terms of the Creative Commons Attribution 4.0 International license.

Surprisingly, depletion of *HSP90* showed a enrichment profile distinct from that observed upon pharmacological inhibition with Hsp90 in C. albicans, for which transcripts involved in responses to oxidative stress were upregulated and transcripts involved in ribosomal and mitochondrial processes were downregulated (see Fig. S5 and Table S2). Compared to Hsp90 depletion, only 744 DEGs in C. albicans showed concordant changes in expression under both conditions (Fig. 6A). We postulate that this was in part a consequence of the fact that depletion of Hsp90 results in cellular consequences different from those resulting from locking the available Hsp90 pool in a particular state in its chaperone cycle (35). Regardless, as both depletion and pharmacological inhibition of Hsp90 induce filamentous growth in C. albicans, we focused our analysis on those DEGs that overlapped under the different experimental conditions. For C. albicans, GO slim mapping of the DEGs identified 249 and 317 transcripts involved in filamentous growth upon Hsp90 inhibition and depletion, respectively (Fig. 6B; see also Table S3). Of these, 107 transcripts showed an overlap under the two conditions, including known regulators of filamentation *HWP1*, *ECE1*, *RBT1*, *BRG1*, *UME6*, *EFH1*, and *BCR1*, which were upregulated, and *YWP1*, which was downregulated (Fig. 6A; see also Table S1). This analysis validates the idea that a core transcriptional response accompanies C. albicans morphogenesis in response to perturbation of Hsp90 function and provides a powerful platform for comparative analyses with the transcriptional response in C. auris.

10.1128/mBio.02529-18.9 TABLE S3 GO slim mapping of differentially expressed genes in response to Hsp90 inhibition and *HSP90* depletion in C. albicans and C. auris. Download Table S3, XLSX file, 0.1 MB. Copyright © 2019 Kim et al. 2019 Kim et al. This content is distributed under the terms of the Creative Commons Attribution 4.0 International license.

Similarly to C. albicans, pharmacological inhibition and genetic depletion of Hsp90 in C. auris induced distinct transcriptional responses, with only 292 DEGs showing concordant changes in expression (Fig. 6A). GO term enrichment analysis of the DEGs in response to Hsp90 inhibition identified upregulation of transcripts involved in ribosomal processes and downregulation of transcripts involved in metabolic and biosynthetic processes (Fig. S6 and Table S2). This is reminiscent of the response of C. albicans to Hsp90 inhibition. Depletion of *HSP90* led to the upregulation of transcripts predicted to be involved in transmembrane transport and downregulation of genes annotated as involved in translation and peptide metabolic processes (Fig. S6 and Table S2). GO slim mapping of the DEGs identified 79 and 33 transcripts involved in filamentous growth upon Hsp90 inhibition and depletion, respectively, but none of these showed an overlap with the known regulators of C. albicans filamentation identified above (Fig. 6B; see also Table S3). Of these, only 10 transcripts showed an overlap under the two conditions (Fig. 6B).

10.1128/mBio.02529-18.6 FIG S6 GO term enrichment of differentially regulated transcripts in response to Hsp90 perturbation in C. auris. Data represent GO term enrichment of C. auris transcripts generated by ClusterProfiler under the following conditions: (A) Hsp90 inhibition by geldanamycin and (B) doxycycline-mediated transcriptional repression of *HSP90* depletion in the *tetO-HSP90* strain. The *y*-axis data show enriched GO terms, and the *x*-axis data show gene ratios. Adjusted *P* value (p.adjust) data are represented by the heat map, and the number of transcripts in each GO term (count) is represented by the size of each of the dots. Download FIG S6, TIF file, 1.4 MB. Copyright © 2019 Kim et al. 2019 Kim et al. This content is distributed under the terms of the Creative Commons Attribution 4.0 International license.

Finally, we identified orthologs of C. auris DEGs from the C. albicans genome in order compare the changes that were common between the two organisms. *HSP70* was one of the common transcripts upregulated upon Hsp90 inhibition and depletion in both organisms (Table S1), consistent with induction of *HSP70* by activation of the Hsf1 heat shock transcription factor upon Hsp90 inhibition (36). Interestingly, the C. auris genome does not contain orthologs of *HWP1* or *ECE1* (Table S1) (18). Further, only three genes annotated as involved in filamentous growth, *ERG3*, *ERG1*, and *DSE1*, were found to be downregulated in both species upon perturbation of Hsp90. Instead, genes orthologous to C. albicans adhesins or adhesin-like proteins such as *ALS1* (B9J08_004112), *IFF4* (B9J08_004451), *PGA26* (B9J08_000117) and to other cell surface proteins such as *RBT1* (B9J08_001458), *PHR1* (B9J08_000918), and *KRE1* (B9J08_005473) were upregulated upon filamentation in C. auris (Table S1). Many of these genes (*ALS1*, *IFF4*, *RBT1*, and *KRE1*) were also upregulated upon Hsp90 inhibition and depletion in C. albicans (Fig. 6C). In addition, genes involved in iron metabolism, such as *FTH1* (B9J08_000170), *FRE9* (B9J08_000168), and *FRP1* (B9J08_004468), were upregulated in both organisms under conditions of filamentous growth, suggesting that iron acquisition might be an important facet of the morphogenetic transition in C. auris, as it is in C. albicans (37). Finally, the ortholog of *YWP1*, encoded by B9J08_001409, was downregulated in response to filamentation in C. auris (Fig. 6A). In total, 28 transcripts were upregulated and 17 transcripts were downregulated upon Hsp90 perturbation in both organisms (Fig. 6C). Although the specific genes that are regulated during the morphogenetic transition are largely distinct between the two species, the regulation of cell surface-associated genes during filamentous growth appears to be conserved between C. auris and C. albicans.

## DISCUSSION

The recent global spread of C. auris suggests that this pathogen has adaptive mechanisms for persistence in hospital environments that remain enigmatic. The adaptive potential of this pathogen is further emphasized by the finding that most C. auris clinical isolates exhibit extremely high levels of resistance to antifungals, particularly the azoles (4). Despite these alarming observations, our understanding of the mechanisms of drug resistance and virulence of this pathogen remain in its infancy. In this study, we examined the impact of C. auris Hsp90 on fluconazole resistance and discovered that this molecular chaperone is important for tolerance of fluconazole, potentially enabling the evolution of drug resistance in otherwise susceptible strains. Further, we identified the Cdr1 ABC transporter as a major contributor to azole resistance in C. auris. Finally, we discovered a novel filamentation program in C. auris that is negatively regulated by Hsp90. Thus, our findings implicate Hsp90 as a central regulator of diverse facets of C. auris biology, including morphogenesis and cellular responses to drug-induced stress.

Our use of the tetracycline-repressible promoter system to regulate the expression of *HSP90* in C. auris establishes a precedent for genetic analysis of essential genes in this pathogen. Although the essentiality of highly conserved genes such as *HSP90* is often maintained across eukaryotes, the essential gene sets can diverge significantly between organisms (38). For example, although there is a correlation between S. cerevisiae and C. albicans with respect to their essential genes, the predictive value from S. cerevisiae to C. albicans is only 52% (38), emphasizing the need to characterize essential genes directly in the organism of interest (38, 39). In addition, antifungal exposure has been shown to elicit distinct transcriptional responses between C. albicans and C. auris, suggesting that distinct genes may contribute to antifungal resistance in C. auris (18). The identification of the essential gene set can lead to the discovery of unique targets for antifungal drug development in emerging pathogens such as C. auris, which might provide a strategy to minimize the use of broad-spectrum antifungals that have limited efficacy against C. auris and to minimize the emergence of resistance.

Hsp90 inhibition reduced the azole tolerance of two C. auris clinical isolates, CDC-382 and CDC-387, and transformed the activity of fluconazole from fungistatic to fungicidal. This suggests that Hsp90 enables key cellular responses to azole-induced cell membrane stress in C. auris such that inhibition of Hsp90 impairs survival. The fluconazole-potentiating effect of Hsp90 inhibition is reminiscent of the results of a study of a series of C. albicans clinical isolates from an AIDS patient, with the early clinical isolates showing an Hsp90-dependent fluconazole tolerance phenotype (8, 27, 28). Over time, these isolates showed stepwise increases in fluconazole resistance that became independent of Hsp90 as the isolates acquired mutations in *ERG11* and in the transcriptional activator of *CDR1*, *TAC1* (8, 27, 28). As CDC-382 and CDC-387 do not contain any known Erg11 substitutions associated with azole resistance and show relatively low levels of *CDR1* expression, these two isolates may reflect a C. auris state prior to fluconazole exposure and acquisition of mutations associated with resistance (6). Hsp90 was dispensable for the resistance of several C. auris strains that harbored mutations in *ERG11* and high levels of *CDR1* expression. This highlights that azole resistance in these strains is likely due to the poor engagement of fluconazole with its cellular target Erg11 and/or to the upregulation of azole efflux pumps, thereby minimizing drug-induced cellular stress.

Although morphogenesis is an important virulence trait in C. albicans and other fungal pathogens (40), the role of filamentous growth in C. auris is unclear. Only one study has reported filamentous growth in C. auris to date, and that growth occurred under conditions of high salt concentrations (31). Here, we showed that the impairment of either Hsp90 function or cell cycle progression also leads to filamentous growth in this pathogen. However, exposure to other canonical C. albicans filament-inducing cues had no impact on the yeast-to-filament transition in C. auris. We performed global analysis of the transcriptional changes upon inhibition or depletion of Hsp90 in C. auris and in C. albicans to characterize conservation and divergence in this morphogenetic program. In C. albicans, filamentation caused by perturbations in Hsp90 was associated with upregulation of filament-associated genes such as *HWP1*, *ECE1*, and *RBT1*, consistent with previous reports (21, 41). In addition, *YWP1*, which encodes a secreted cell wall protein specific to yeast growth (42), was downregulated, further supporting the idea of a filamentous growth program caused by Hsp90 perturbation. The transcriptional changes in C. auris were reminiscent of those observed in C. albicans. Despite the lack of a clear *HWP1* ortholog in C. auris, predicted cell surface proteins such as *PHR1* (B9J08_003910), *RBT1* (B9J08_001458), and *PGA26* (B9J08_000117) were highly upregulated and an ortholog of *YWP1*, B9J08_001409, was downregulated. In C. albicans, filamentous growth is also associated with extensive cell surface remodeling as indicated by changes in cell surface components at the transcriptomic and proteomic levels (32). Although universal changes in cell surface composition upon morphogenesis have not been identified in fungi, cell surface proteins are often important regulators of morphogenesis and many are required for flocculation and adhesion, which are phenotypes associated with specific morphogenetic states (43). While it is clear that the environmental cues that trigger C. auris morphogenesis are largely distinct from those that trigger C. albicans morphogenesis, our results suggest that transcriptional remodeling of the cell surface is conserved during this developmental transition. Cell surface genes may be required for proper formation of filaments, similarly to the results seen with *FLO11* in S. cerevisiae, or may be important for modulating host immune responses, as is the case in C. albicans, where active remodeling occurs upon phagocytosis by macrophages, which then drives macrophage programmed cell death (38, 44). Further exploration of the cues that induce C. auris morphogenesis and the genetic circuitry involved is poised to uncover key facets of the biology of the pathogen.

Taking the results together, our work establishes a powerful system to study essential genes in an emerging pathogen, identifies strategies to modulate drug tolerance and resistance, and highlights conservation and divergence in a developmental program induced by perturbation of protein homeostasis in fungal pathogens.

## MATERIALS AND METHODS

### Growth conditions.

All strains were archived in 25% glycerol and stored at −80°C. Strains were grown in YPD (1% yeast extract, 2% Bacto peptone, 2% glucose) agar at 30°C unless otherwise specified. For solid media, 2% agar was added.

### Strain construction.

Strains used in this study are listed in Table S4 in the supplemental material. To prepare cultures for electroporation, 5 μl of a saturated overnight culture was diluted into 200 ml of fresh YPD and incubated at 30°C with shaking until the optical density at 600 nm (OD_600_) was between 1.6 and 2.2. Cells were pelleted and resuspended in 40 ml of 1× TE (10 mM Tris, 1 mM EDTA) buffer and 0.1 M lithium acetate and incubated at 30°C with shaking. After 1 h, 1 ml of 1 M dithiothreitol (DTT) was added and the reaction mixture was incubated at 30°C with shaking for 30 min. The cells were pelleted and washed 2 times with ice-cold sterile water. The cells were then pelleted and washed once with ice-cold 1 M sorbitol and finally resuspended in 200 μl of 1 M sorbitol.

10.1128/mBio.02529-18.10 TABLE S4 Strains, oligonucleotides, and plasmids used in this study. Download Table S4, DOCX file, 0.1 MB. Copyright © 2019 Kim et al. 2019 Kim et al. This content is distributed under the terms of the Creative Commons Attribution 4.0 International license.

A 40-μl volume of electrocompetent cells was mixed with 3 mg of repair cassette DNA and 1 mg of Cas9-sgRNA cassette DNA in a 0.2-cm-path-length cuvette. All DNA used for electroporation was purified by ethanol precipitation. The digested Cas9-sgRNA cassette or amplified repair cassette was mixed with 2 volumes of anhydrous ethanol and 0.1 volume of 3 M sodium acetate (pH 5.0). Mixtures were incubated at −20°C for a minimum of 20 min and then pelleted at 4°C. DNA was washed 3 times with 70% ethanol, dried completely, and then resuspended in sterile water.

The electroporation settings were as follows: 1.8 kV, 200 Ω, and 25 μF. After electroporation, the cuvettes were filled with ice-cold 1 M sorbitol to reach a volume of 1 ml. The cells were pelleted, resuspended in 10 ml of YPD, and incubated at 30°C for 4 h. The cells were plated on YPD agar plus 150 μg/ml nourseothricin (NAT) (Jena Bioscience) or YPD agar plus 150 μg/ml NAT plus 600 μg/ml hygromycin B (HygB) (Bioshop) and incubated at 30°C for 48 h.

### Plasmid construction.

Cloning procedures were performed following standard protocols. Transformed DH5α competent Escherichia coli cells (Invitrogen) were grown on LB with 2% agar (Sigma) containing either 100 μg/ml ampicillin (AMP) (Bioshop) or 100 μg/ml AMP plus 50 μg/ml NAT and incubated at 37°C overnight. Plasmids used in this study are listed in Table S4. The absence of nonsynonymous mutations in plasmids was verified by sequencing. Primers used in this study are listed in Table S4.

### Genomic DNA preparation.

A 1-ml volume of overnight cultures were transferred to screw-cap tubes containing acid-washed glass beads, 200 μl of phenol:chloroform:iso-amyl alcohol, and 200 μl of breaking buffer (2% Triton X-100, 1 mM EDTA, 1% SDS, 100 mM NaCl, 10 mM Tris, pH 8.0) and was subjected to vigorous vortex mixing for 2 min. The tubes were centrifuged, and the supernatant was transferred to tubes containing equal volume of chloroform and gently mixed. The tubes were centrifuged, and the supernatant was transferred to tubes containing 2.5 volumes of anhydrous ethanol and 0.1 volume of 3 M sodium acetate (pH 5.0). The tubes were subjected to gentle mixing and placed at −20°C for at least 20 min. The tubes were then centrifuged at 4°C for 30 min and, the DNA pellets were washed with 70% ethanol and then completely dried. The DNA was resuspended in sterile water.

### RNA preparation.

Overnight cultures were inoculated into 10 ml YPD with doxycycline (0.5 μg/ml) or without doxycycline to reach the final OD_600_ of 0.1 and were incubated at 30°C with shaking for 24 h. Subsequently, cultures were further subcultured into 25 ml of YPD without or with doxycycline (5 μg/ml) to reach a final OD_600_ of 0.1 and incubated at 30°C with shaking for 4 h. For geldanamycin treatment, the overnight cultures were inoculated into 10 ml YPD with geldanamycin (10 μM for C. albicans, 80 μM for C. auris) or without geldanamycin to reach a final OD_600_ of 0.1 and were incubated at 30°C with shaking for 4 h. The cells were pelleted, washed with ice-cold sterile water, flash frozen, and stored at −80°C for at least 24 h. For quantitative reverse transcription-PCR (qRT-PCR), RNA was isolated using an RNeasy kit (Qiagen) and treated with an RNase-free DNase set (Qiagen). For RNA-Seq, RNA was isolated using an RNeasy minikit (Qiagen) and treated using a DNA Free kit (Ambion).

### Quantitative RT-PCR.

cDNA was synthesized using an AffinityScript multitemperature cDNA synthesis kit (Agilent Technologies). Quantitative PCR (qPCR) was performed using FastSYBR green Master Mix (Applied Biosystems) and a Bio-Rad CFX384 real-time system under the following cycling conditions: 95°C for 3 min, 95°C for 10 s, and 60°C for 30 s for 40 cycles. Data were analyzed using Bio-Rad CFX Manager 3.1, and all data were normalized to C. auris *ACT1* and *GPD1*.

### Spotting assay.

Saturated overnight cultures were diluted 1,000-fold in sterile water. Serial dilutions (100-fold) were subsequently made in sterile water. A 5-μl volume of the diluted cultures was spotted onto YPD plates in the absence and presence of doxycycline (50 μg/ml). The plates were incubated at 30°C for 2 days before imaging was performed.

### Etest fluconazole susceptibility assay.

Overnight cultures were counted using a hemocytometer and diluted to 5 × 10^6^ cells/ml in sterile water, and 200 μl was plated onto YPD plates without and with doxycycline (0.1 μg/ml or 10 μg/ml) to reach the final cell number of 1 × 10^6^ cells. Fluconazole Etest strips (bioMérieux) were placed after drying the plates. Plates were incubated at 30°C for 48 h before imaging was performed.

### Drug susceptibility assay.

Approximately 1 × 10^3^ cells were inoculated with a 2-fold gradient matrix of fluconazole (Carbosynth) or of geldanamycin (LC Laboratories) or of a combination of the two, as indicated, in 96-well microtiter plates to reach the final volume of 200 μl in YPD. The plates were incubated at 30°C for 48 h before measurement of the OD_600_ was performed using SpectraMax M2e (Molecular Devices). The relative growth values were calculated by normalizing OD_600_ values against the no-compound control and were plotted as a heat map using Java TreeView.

### Filamentation assay.

Overnight cultures were diluted to the final OD_600_ of 0.1 in 10 ml YPD, YPD with 10% serum, YPD with geldanamycin (10 μM for C. albicans and 80 μM for C. auris), YPD with hydroxyurea (Bioshop), YPD with doxycycline (5 μg/ml), Spider medium (45), or RPMI 1640 (10.4 g/l RPMI 1640 powder, 3.5% morpholinepropanesulfonic acid [MOPS], 2% glucose, 5 mg/ml histidine, pH 7.0) and incubated at 30°C, 37°C, or 42°C for 6 h as indicated. Cells were imaged using differential interference contrast (DIC) microscopy (Zeiss Axio Imager).

### RNA sequencing.

RNA was prepared in quadruplicate as described above. Library preparation was carried out using a TruSeq stranded mRNA sample preparation kit according to the manufacturer’s instructions. Paired-end (100) sequencing was done using Illumina sequencing technology. Data are available on the NCBI SRA database (SRP173838). FastQC was used for the quality checking of the raw fastq data (46). These raw reads were aligned against the C. auris B8441 reference genome (GenBank assembly accession no. GCA_002759435.2) (18) using HISAT2 (47). StringTie and the prepDE.py Python script provided with the StringTie tool were used to assemble the alignments into transcripts and to extract the raw read counts for reference genomic features, respectively (48, 49). Raw read counts were processed using the DESeq2 package for differential gene expression analysis (50). Genes with a *q* value of ≤0.05 and a log2(fold change) value of ≥0.58 were considered to represent significantly differentially expressed genes (DEGs). Gene annotation of the C. auris B8441 genome was performed using RNA-Seq paired-end reads to improve gene calling and structure predictions (18). Genes containing PFAM domains found in repetitive elements or overlapping tRNA/rRNA features were removed. Genes were functionally annotated using GO terms and Blast2GO CLI v.1.3.3 (51). GO term enrichment of significant DEGs was performed using ClusterProfiler (52). C. auris gene orthologs to C. albicans were assigned using OrthoMCL (53). Additionally, blastx was used to annotate the C. auris genes which did not map to any C. albicans ortholog (54).
