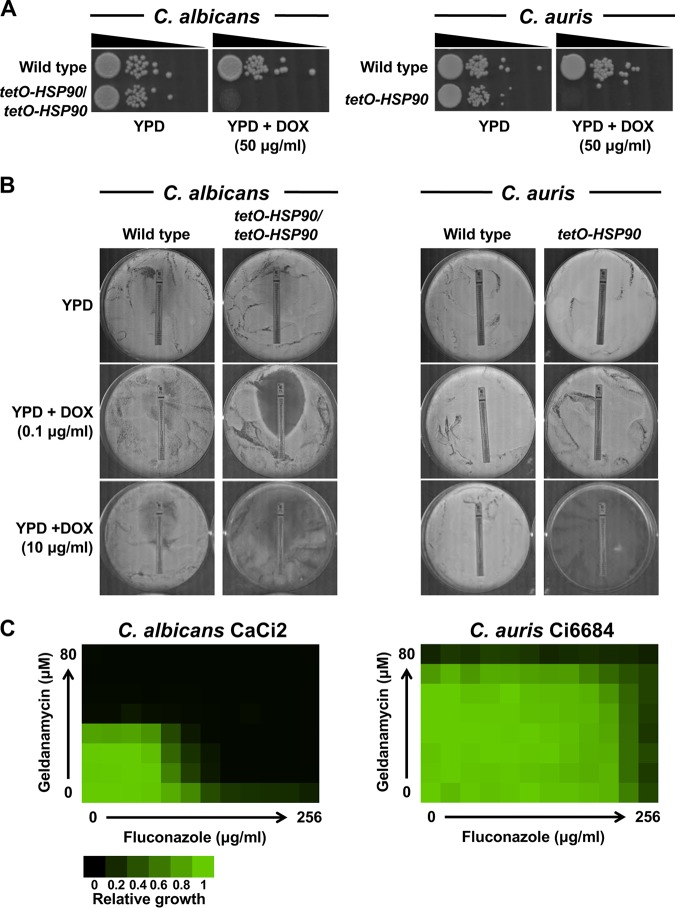# Erratum for Kim et al., “Genetic Analysis of Candida auris Implicates Hsp90 in Morphogenesis and Azole Tolerance and Cdr1 in Azole Resistance”

**DOI:** 10.1128/mBio.00346-19

**Published:** 2019-03-19

**Authors:** Sang Hu Kim, Kali R. Iyer, Lakhansing Pardeshi, José F. Muñoz, Nicole Robbins, Christina A. Cuomo, Koon Ho Wong, Leah E. Cowen

**Affiliations:** a Department of Molecular Genetics, University of Toronto, Toronto, Ontario, Canada; b Genomics and Bioinformatics Core, Faculty of Health Sciences, University of Macau, Macau, China; c Broad Institute of Harvard and Massachusetts Institute of Technology, Cambridge, Massachusetts, USA; d Faculty of Health Sciences, University of Macau, Macau, China; e Institute of Translational Medicine, University of Macau, Macau, China

## ERRATUM

Volume 10, no. 1, e02529-18, 2019, https://doi.org/10.1128/mBio.02529-18. Figure 1A spotting images were incorrectly duplicated for the *Candida albicans* and *Candida auris* plates. We confirmed the essentiality of *HSP90* expression in *C. albicans* and *C. auris* with multiple biological replicates and multiple assays, as in Fig. 1B (repression of the *HSP90* transcript in a *tetO*-*HSP90* strain using 10 μg/ml DOX) and in Fig. 1C and Fig. S3 in the supplemental material (pharmacological inhibition of Hsp90 using geldanamycin). Figure 1A has been updated to include the correct spotting images of *C. auris* strains to demonstrate the essentiality of *HSP90* expression.

**FIG 1:**