# Supplementary figures and images for: Genetic Analysis of Candida auris Implicates Hsp90 in Morphogenesis and Azole Tolerance and Cdr1 in Azole Resistance
Source: mBio. 2019 Jan 29;10(1):e02529-18. doi: 10.1128/mBio.02529-18 (PMC6355988; doi:10.1128/mBio.02529-18)

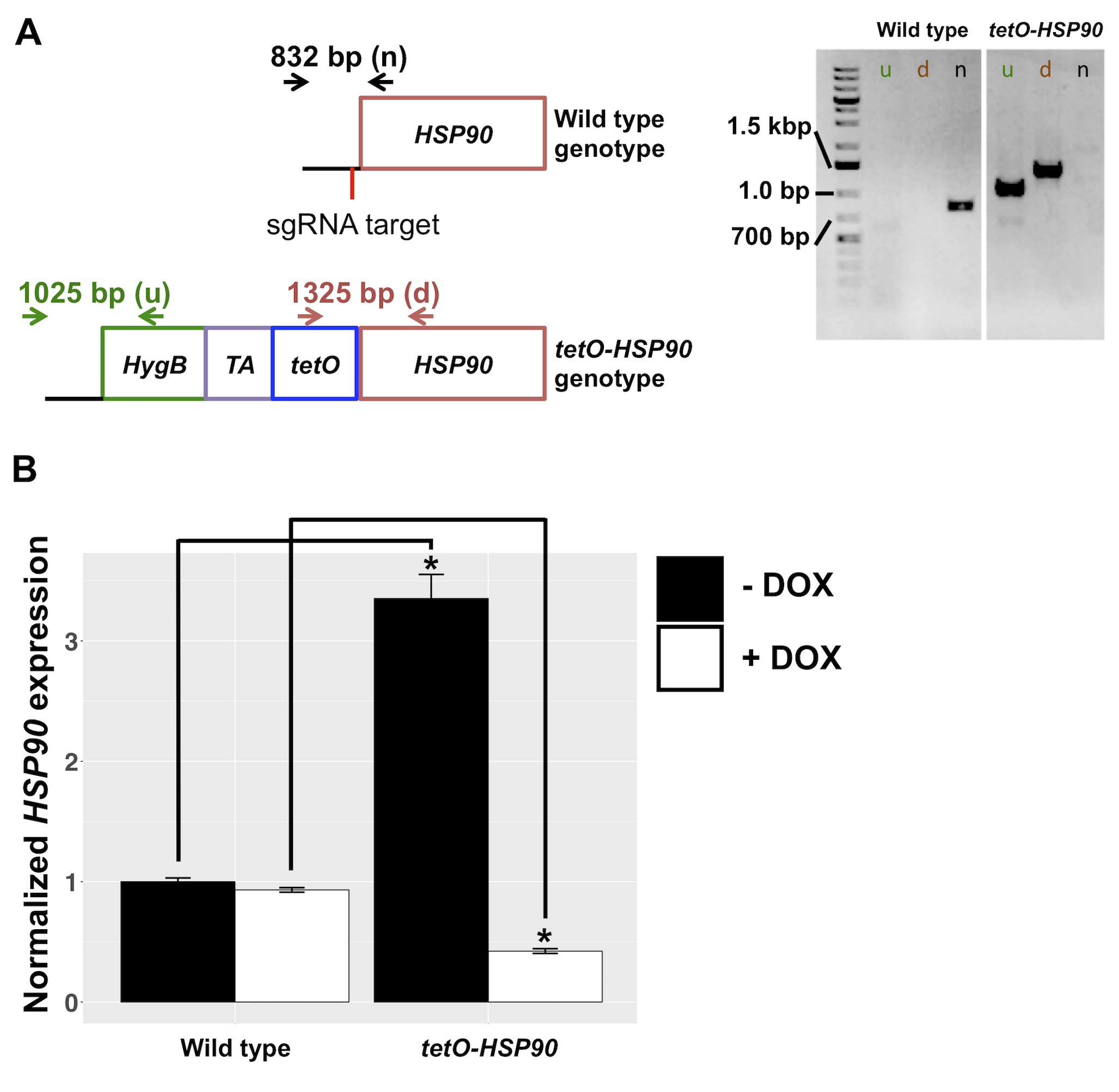

Supplement: FIG S1 [file mBio.02529-18-sf001.tif]

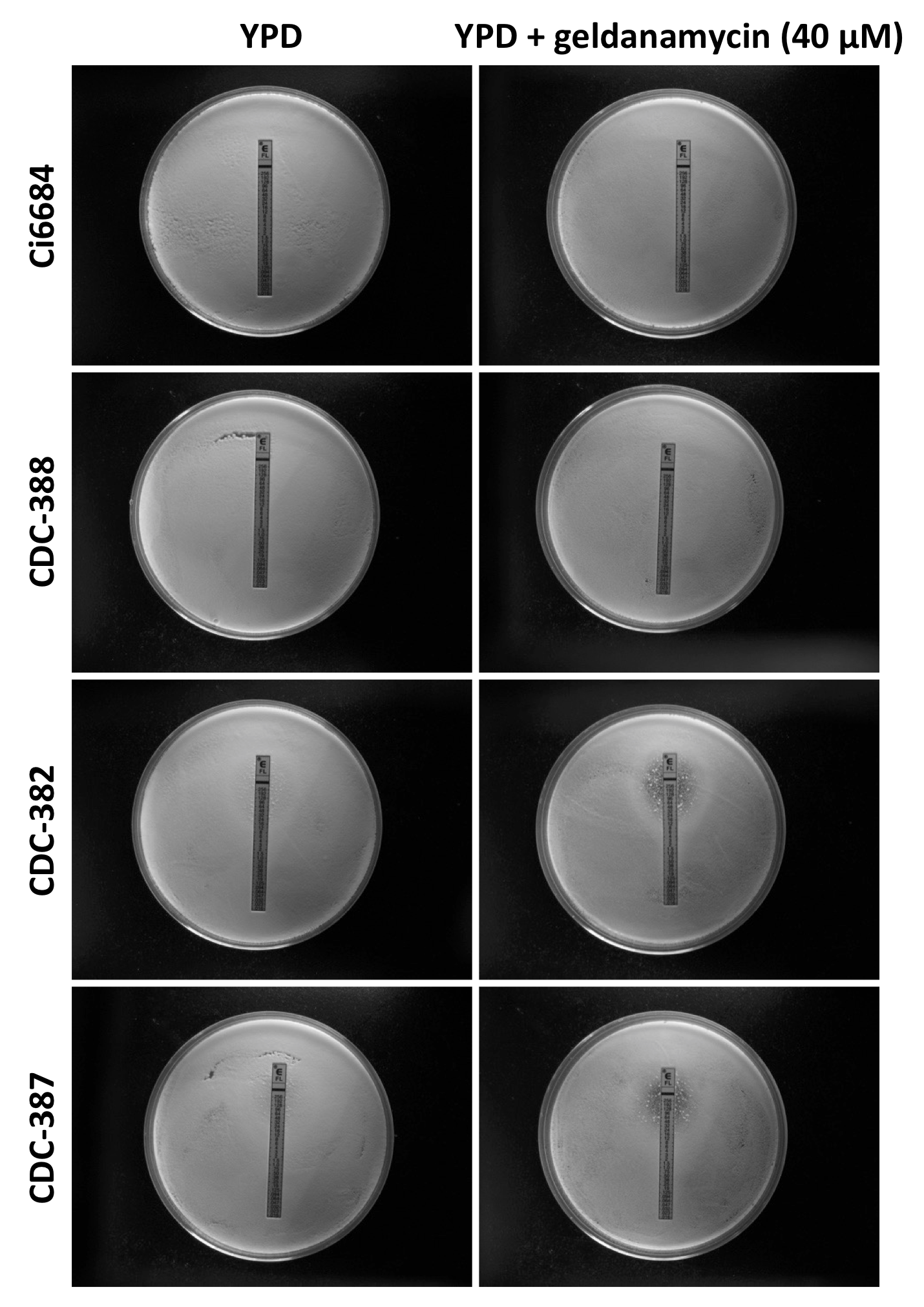

Supplement: FIG S2 [file mBio.02529-18-sf002.tif]

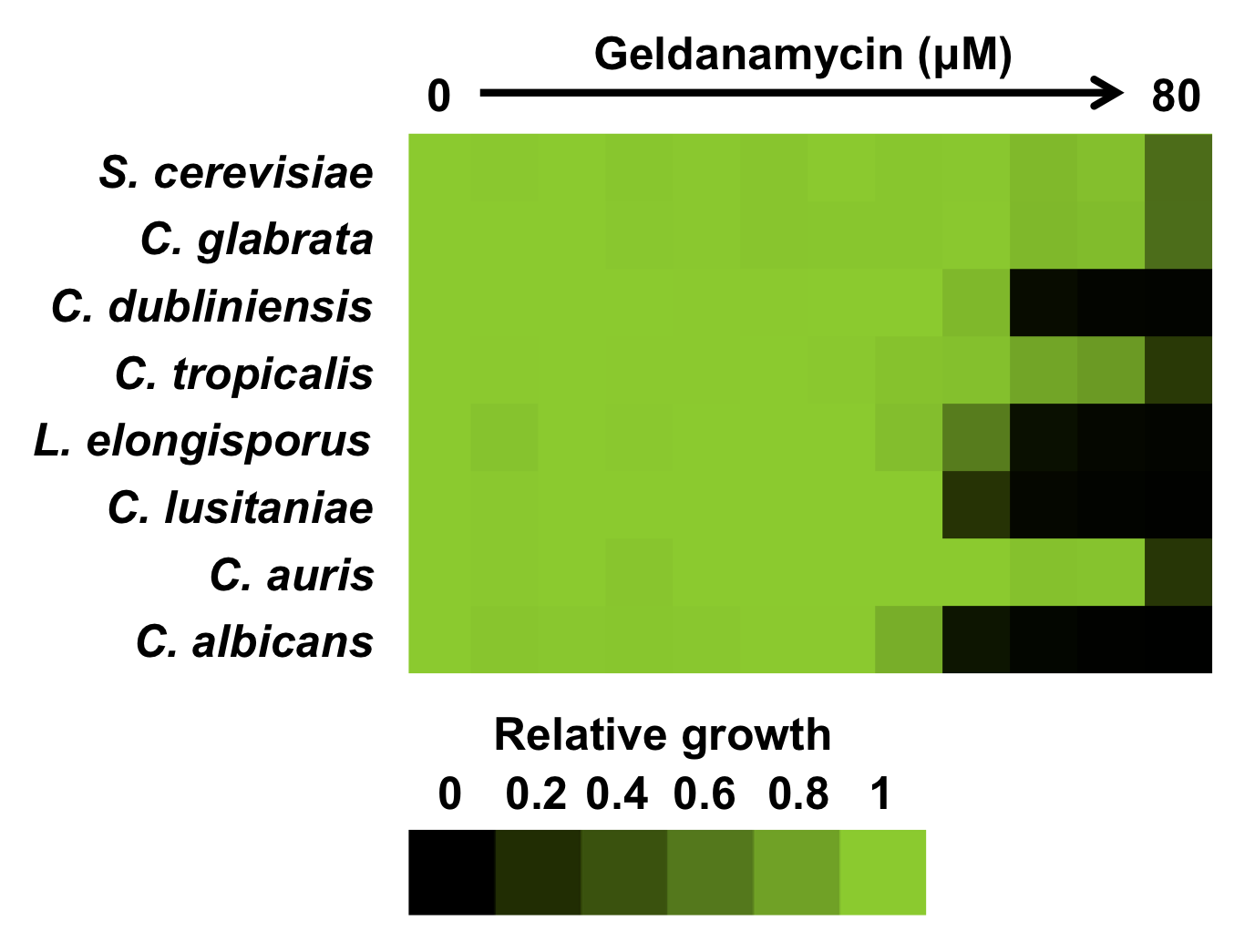

Supplement: FIG S3 [file mBio.02529-18-sf003.tif]

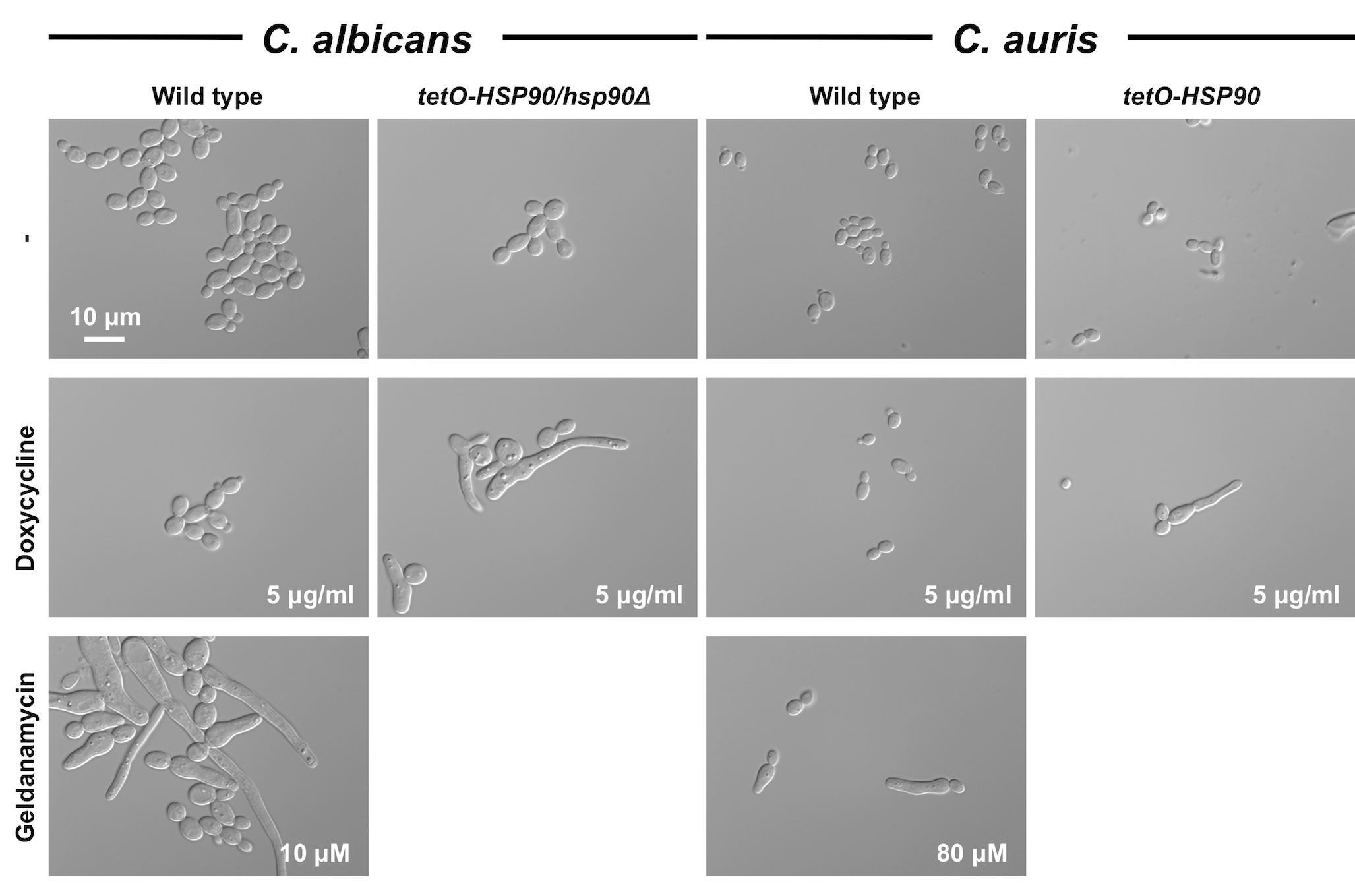

Supplement: FIG S4 [file mBio.02529-18-sf004.tif]

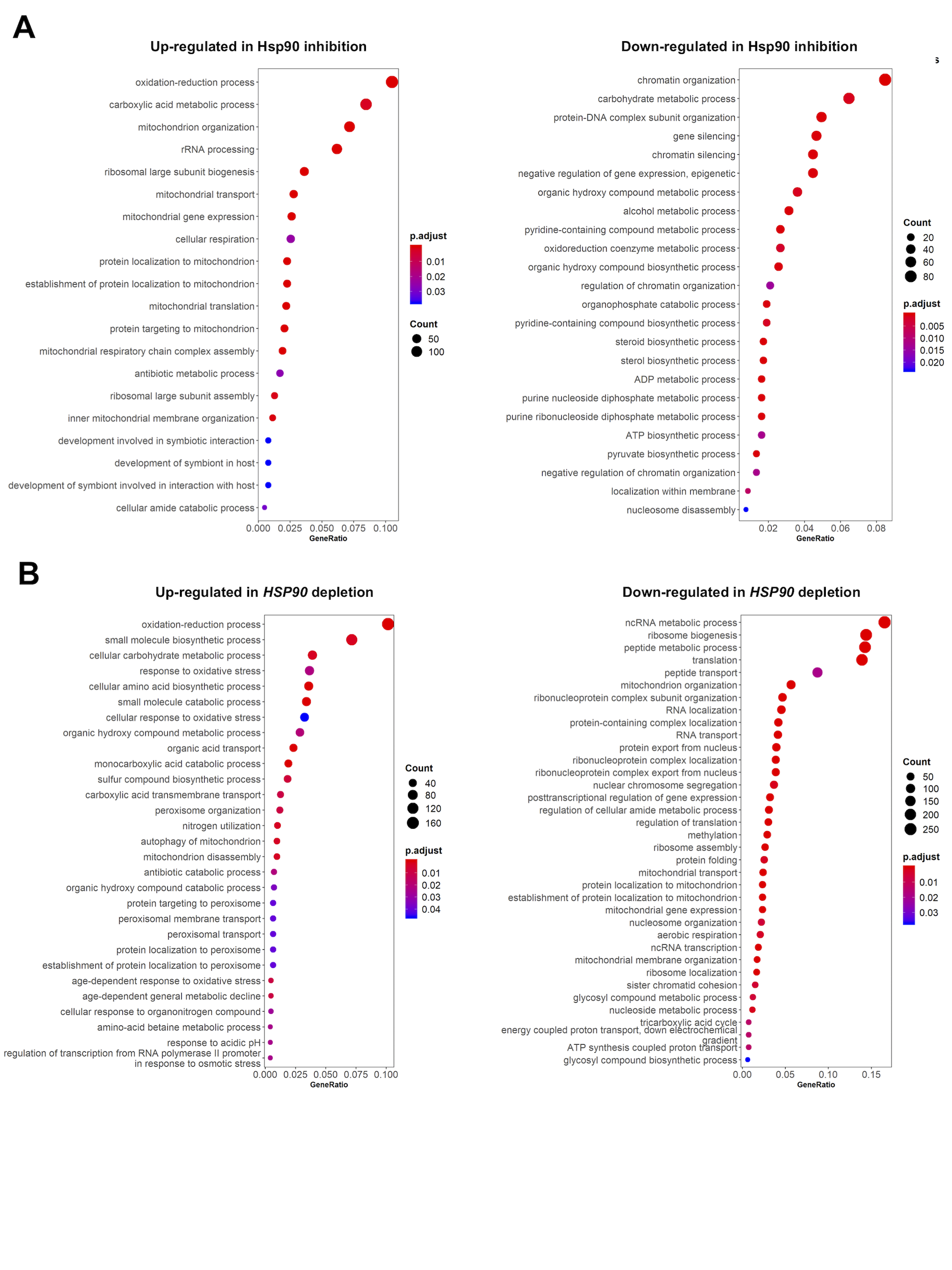

Supplement: FIG S5 [file mBio.02529-18-sf005.tif]

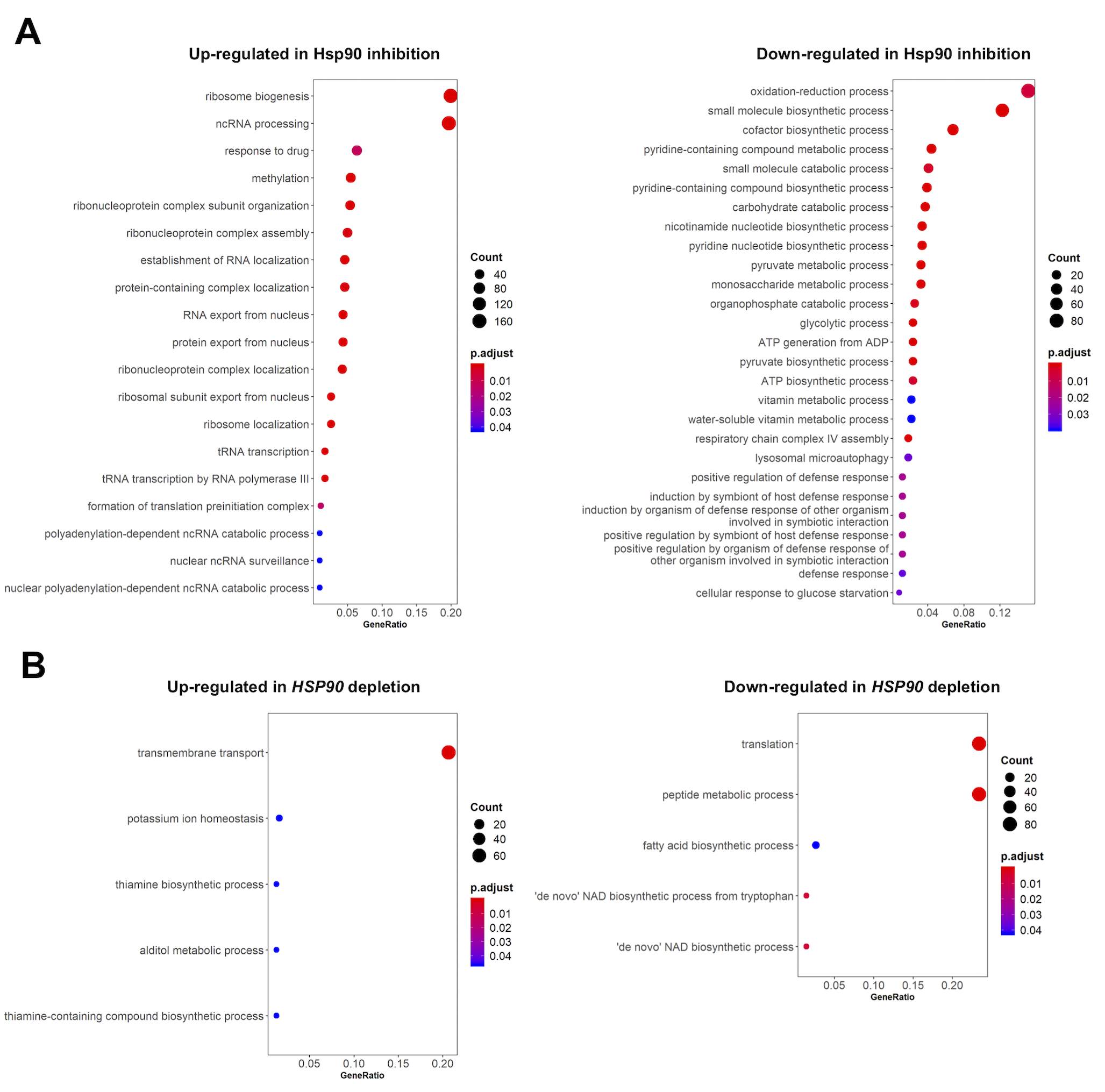

Supplement: FIG S6 [file mBio.02529-18-sf006.tif]
