# Supplementary material for: Genetic Analysis of Candida auris Implicates Hsp90 in Morphogenesis and Azole Tolerance and Cdr1 in Azole Resistance
Source: mBio. 2019 Jan 29;10(1):e02529-18. doi: 10.1128/mBio.02529-18 (PMC6355988; doi:10.1128/mBio.02529-18)
Supplement: TABLE S4 [file mBio.02529-18-st004.docx]

**Supplementary table 4: Reagents used in this study**

**Strains used in this study.**

| Strain ID | Description | | Genotype | Source |
| --- | --- | --- | --- | --- |
| CaLC5083 | *C. auris* Ci6684 | | Clinical isolate | (1) |
| CaLC5147 | *C. auris* Ci6684 *tetO-ADE2* | | *HygB-TAR-tetO-ADE2 NAT* | This study |
| CaLC5218 | *C. auris* Ci6684 *tetO-HSP90* | | *HygB-TAR-tetO-HSP90 NAT* | This study |
| CaLC5280 | *C. auris* Ci6684 *cdr1Δ* | | *cdr1Δ* | This study |
| CaLC239 | *C. albicans* WT | | *arg4Δ/arg4Δ, his1Δ/his1Δ, URA3/ura3Δ::imm434 IRO1/iro1Δ::imm434* | (2) |
| CaLC4855 | *C. albicans tetO-HSP90/tetO-HSP90* | | *arg4Δ/arg4Δ, his1Δ/his1Δ, URA3/ura3Δ::imm434 IRO1/iro1Δ::imm434, TAR-tetOp-HSP90/TAR-tetOp-HSP90* | (3) |
| CaLC3385 | *C. albicans* *tetO-HSP90/hsp90Δ* | | *arg4Δ/arg4Δ,his1Δ/his1Δ, URA3/ura3Δ::imm434 IRO1/iro1Δ::imm434,hsp90::FRT, CaTAR-FRT::TetO-HSP90, HSF1/HSF1-TAP-ARG4* | (3) |
| CaLC79 | CaCi2 | | Clinical isolate | (4) |
| CdLC268 | *C. dubliniensis* MYA-577 | | Clinical isolate | (5) |
| CtLC575 | *C. tropicalis* 1685 | | Clinical isolate | Heitman Lab |
| LeLC578 | *L. elongisporus* NRLLYB-4239 reference strain | | Clinical isolate | Heitman Lab |
| ClLC572 | *C. lusitaniae* ATCC 42720 reference strain | | Clinical isolate | Heitman Lab |
| ScLC382 | *S. cerevisiae* Sigma | | *ura3Δ* | Kohler Lab |
| CgLC1002 | *C. glabrata* BG2 | | Clinical isolate | Cormack Lab |
| CDC-0381 | | CDC_B11220 | Clinical isolate | CDC and (6) |
| CDC-0382 | |  | Clinical isolate | CDC and (6) |
| CDC-0383 | | CDC_B11221 | Clinical isolate | CDC and (6) |
| CDC-0384 | | CDC_B11222 | Clinical isolate | CDC and (6) |
| CDC-0385 | | CDC_B11244 | Clinical isolate | CDC and (6) |
| CDC-0386 | | CDC_B11245 | Clinical isolate | CDC and (6) |
| CDC-0387 | | CDC_B8441 | Clinical isolate | CDC and (6) |
| CDC-0388 | | CDC_B11098 | Clinical isolate | CDC and (6) |
| CDC-0389 | |  | Clinical isolate | CDC and (6) |
| CDC-0390 | |  | Clinical isolate | CDC and (6) |

**CaLC5218: *C. auris tetO-HSP90***

The HygB-TAR*-tetO* repair cassette with homology to the *HSP90* promoter and open reading frame was generated by fusion PCR. Promoter homology was amplified from CaLC5083 gDNA using oLC5832/oLC5833 (887 bp). Open reading frame homology was amplified from CaLC5083 gDNA using oLC5836/oLC5837 (1,115 bp). The HygB-TAR*-tetO* cassette was amplified from pLC1031 using oLC5834/oLC5835 (5,138 bp). The three amplicons were fused using oLC5883/oLC5884 (6,796 bp). pLC1025 was double digested with KpnI/SacI to liberate the Cas9-sgRNA cassette targeting *HSP90* promoter. The Cas9-sgRNA and repair cassettes were used to transform wild-type *C. auris* (CaLC5083) by electroporation. This allows ectopic integration and expression of Cas9 and sgRNA targeting *HSP90* promoter Integration of the HygB-TAR*-tetO* cassette to *HSP90* locus was verified by amplifying upstream integration (oLC5832/oLC5959, 1,025 bp), downstream integration (oLC4714/oLC5837, 1,325 bp), and absence of the native promoter (oLC5958/oLC5957, 832 bp).

**CaLC5280: *C. auris* *cdr1Δ***

The NAT cassette with homology to the *CDR1* promoter and terminator was generated by fusion PCR. Promoter homology was amplified from CaLC5083 gDNA using oLC6020/oLC6305 (994 bp). Terminator homology was amplified from CaLC5083 gDNA using oLC6306/oLC6025 (890 bp). The NAT cassette was amplified from pLC1049 using oLC6296/oLC6304 (1,213 bp). The three amplicons were fused using oLC6024/oLC6307 (2,851 bp). This cassette was used to transform wild-type *C. auris* (CaLC5083) by electroporation. Integration of the NAT cassette to the *CDR1* locus was verified by amplifying upstream integration (oLC6221/oLC6308, 1,347 bp), downstream integration (oLC274/oLC6023, 1,060 bp), and absence of the native *CDR1* (oLC6231/oLC6169, 524 bp).

**Plasmids used in this study.**

| Plasmid ID | Description | Source |
| --- | --- | --- |
| pV1200 | CaCas9/sgRNA plasmid | (7) |
| pLC1015 | pV1200 + Cau*ENO1* + Cau*SNR52p* | This study |
| pLC1025 | pLC1015-Cau*HSP90p* | This study |
| pLC982 | pYM70 Ca*HygB* | (8) |
| pUC19 | pUC19 |  |
| pLC1031 | *HygB*-*TAR*-*tetO* | This study |
| pLC605 | *NAT*-FLP-*TAR*-*tetO* | (3) |
| pLC1049 | *NAT*-*TAR*-*tetO* | This study |
| pLC1025 | *C. auris* *HSP90p* Cas9-sgRNA | This study |

pLC1015: pV1200 + Cau*ENO1* + Cau*SNR52*p

The CRISPR/Cas9 system was adapted for use in *C. auris* by modifying pV1200 from the *C. albicans* CRISPR/Cas9 system(7). The *C. auris* *ENO1* promoter was amplified from gDNA using oLC5451/oLC5452 (392 bp) and digested with KpnI/XmaI. The *C. auris* *ENO1* terminator was amplified from gDNA using oLC5453/oLC5454 (458 bp) and digested with SacII/SacI. The two amplicons were sequentially cloned into pV1200 by KpnI/XmaI digestion followed by SacII/SacI digestion. The integration of *C. auris* *ENO1* promoter was verified by colony PCR using oLC5395/oLC5399 (554 bp). The integration of *C. auris* *ENO1* terminator was verified by colony PCR using oLC4609/oLC5398 (772 bp). The *C. auris* *SNR52* promoter was amplified from gDNA using oLC5574/oLC5553 (662 bp). sgRNA hairpin sequence was amplified from pV1200 using oLC5554/oLC5555 (178 bp). The two amplicons were fused using oLC5574/oLC5555 (830 bp) and digested with NotI/SacII. This was inserted into the NotI/SacII digested plasmid and the integration was verified by colony PCR using oLC274/oLC5555 (830 bp). The final product was verified by Sanger sequencing.

pLC1031: HygB-TAR-*tetO*

The HygB resistance cassette was amplified from pLC982 using oLC5579/oLC5580 (2,074 bp). The tetracycline repressible transactivator (TAR) and activator binding sequence (*tetO*) were amplified from CaLC3786 gDNA using oLC5581/oLC5582 (3,004 bp). The two amplicons were fused using oLC5579/oLC5582 (5,098 bp) and digested with SphI/XmaI. This was inserted into SphI/XmaI digested pUC19 and the upstream and downstream integration were verified by colony PCR using oLC249/oLC3834 (1741 bp) and oLC698/oLC1713 (1311 bp) respectively. The final product was sequence verified by Sanger sequencing.

pLC1049: NAT-TAR-*tetO*

A TAR-NAT*-tetO* cassette was amplified from pLC605 using oLC6270/oLC6271 (7,030 bp) and digested with SalI. This was self-ligated and the final product was sequence verified by Sanger sequencing.

pLC1025: *C. auris* *HSP90p* Cas9-sgRNA

pLC1015 was digested with BsmBI and ligated with self-annealed oLC5682/oLC5534. Integration of the sgRNA was verified by Sanger sequencing. The cassette was liberated by digesting with KpnI/SacI.

**Oligonucleotides used in this study.**

| Oligo ID | Description | Sequence (5' to 3') |
| --- | --- | --- |
| oLC5451 | KpnI_Cau*ENO1*-371F | gcatgcggtaccccaggattctacgcgcattg |
| oLC5452 | XmaI_Cau*ENO1*-19R | gcatgccccgggtgatgaaaattaagtttgga |
| oLC5453 | SacII_FRT_*ENO1*+1321F | gcatgcccgcgggaagttcctatactttctagagaataggaacttcacgccgggtttgcgcttcaaaccactt |
| oLC5454 | SacI_*ENO1*+1759R | gcatgcgagctcgcgggagggttggataatct |
| oLC5574 | NotI_Cau*SNR52*-662F | atgcgcggccgcacagactcaatcaacgaagct |
| oLC4609 | Calb*Snr52p*-fwd1 | gaaacttcggcccaataggattgg |
| oLC5395 | KpnI_*ENO1*-371F | atgcggtaccccaggattctacgcgcattg |
| oLC5399 | CaCas9+159R | gcagtttcaccggagtcg |
| oLC5398 | SacI_*ENO1*+1759R | atgcgagctcgcgggagggttggataatct |
| oLC5553 | sgRNA_Cau*SNR52*-1R | cgagacggaattccgtctcctgttttctgctgagggagtc |
| oLC5554 | sgRNA+1F | ggagacggaattccgtctcgttttagagctagaaatagca |
| oLC5555 | SacII_sgRNA+148R | atgcccgcggtggcggcaaaactaattctt |
| oLC5579 | SphI_*TEF2p*F | atgcgcatgcgacgtcgtatagtgcttgct |
| oLC5580 | TARpR_*ACT1t*R | catggtcatagctgtttcccattttatgatggaatgaat |
| oLC5581 | *ACT1*tF_*TARp*F | attcattccatcataaaatgggaaacagctatgaccatg |
| oLC5582 | XmaI_*tetO*R | atgccccgggcgactatttatatttgtatg |
| oLC6270 | SalI-Ca*NAT^R^* | gctagtcgacactggatggcggcgttagta |
| oLC249 | pBS800-R | cagctatgaccatgattacg |
| oLC3834 | Ca*ACT1*+2311-R | ccagatttccagaatttcac |
| oLC698 | OAD+824-F | cgcgtttggaatcactacagg |
| oLC1713 | pLC10+1934F | cacgacgttgtaaaacgacg |
| oLC6271 | SalI-Ca*TAR* | gctagtcgacgacattttatgatggaatga |
| oLC5682 | Cau*HSP90p*_sgRNA_top | aacagatgctgcttgactatgagtag |
| oLC5534 | Cau*HSP90p*_sgRNA_bot | aaaactactcatagtcaagcagcatc |
| oLC4714 | *tetOp*+488F | tcgtttctgatgggcttttc |
| oLC5832 | Cau*HSP90*-894F | gttcacagttggtcagagcc |
| oLC5833 | *HygB*R_Cau*HSP90*-27R | agcaagcactatacgacgtcgactatgagtatggggattg |
| oLC5834 | Cau*HSP90*-27F_*HygB*F | caatccccatactcatagtcgacgtcgtatagtgcttgct |
| oLC5835 | Cau*HSP90*+0R_*tetOp*R | gtttcaaccttctcgggcatcgactatttatatttgtatg |
| oLC5836 | *tetOp*F_Cau*HSP90*+0F | catacaaatataaatagtcgatgcccgagaaggttgaaac |
| oLC5837 | Cau*HSP90*+1095R | tcctcagagtccacaacacc |
| oLC5883 | Cau*HSP90*-768F | gatgggatgttgcaggtgtg |
| oLC5884 | Cau*HSP90*+917R | gaccctcgacagaaaagtgc |
| oLC5959 | *TEF2p*+118R | tgcggcacaattgaataggg |
| oLC5957 | Cau*HSP90*+496R | accacgcttgattctctcgt |
| oLC5958 | Cau*HSP90*-316F | ccttagaggcaccttcacca |
| oLC6293 | pLC605 *TAR* F | ttgagatggagccgtcaaat |
| oLC6294 | pLC605 *tetO* R | cgactatttatatttgtatg |
| oLC534 | Ca*TAR*-797-R | gatggagatagtttacgg |
| oLC6020 | Cau*CDR1*-989F | taacgcaaaaggaccatggc |
| oLC6305 | *NAT*_Cau*CDR1*-35R | ctatactgctgtcgattcgatactaacgccgccatccagtactacatgcgatatatatat |
| oLC6306 | *NAT*_Cau*CDR1*+69F | cgctggccgggtgacccggcggggacgaggcaagcttgattgagctcgtgtgtgtcatca |
| oLC6025 | Cau*CDR1*+5426R | tttctgtctctctgagggca |
| oLC6296 | pLC605 *NAT* F | actggatggcggcgttagta |
| oLC6304 | pLC605 *NAT* R | atcaagcttgcctcgtcc |
| oLC6024 | Cau*CDR1*-925F | cggcccatgataaccctcta |
| oLC6307 | Cau*CDR1*-797R | cccacatttcgagaaaagga |
| oLC6221 | Cau*CDR1*-1298F | acagctggattcgacatggg |
| oLC6308 | pLC605*NAT*_26 | tggtcgctatactgctgtcg |
| oLC6023 | Cau*CDR1*+5493R | cgcccttgataatgtccacg |
| oLC274 | pJK863down-F | ctgtcaaggagggtattctgg |
| oLC6231 | Cau*CDR1*+2175F | tttgtgccttcaggaggacc |
| oLC6169 | Cau*CDR1*+2679R | gcagtgatctgacctggctt |
| oLC5727 | Cau*ACT1*+121F | accccaagtccaacagagag |
| oLC5728 | Cau*ACT1*+316R | tccagccaagtcaagtctca |
| oLC5729 | Cau*GPD1*+141F | atccttgctgaaaacgctgc |
| oLC5730 | Cau*GPD1*+318R | tcctcggccacctttacaat |
| oLC5956 | Cau*HSP90*+287F | taccattgccaagtccggta |
| oLC5957 | Cau*HSP90*+496R | accacgcttgattctctcgt |

**References**

1. Chatterjee S, Alampalli SV, Nageshan RK, Chettiar ST, Joshi S, Tatu US. 2015. Draft genome of a commonly misdiagnosed multidrug resistant pathogen *Candida auris*. BMC Genomics 16:686.

2. Noble SM, Johnson AD. 2005. Strains and strategies for large-scale gene deletion studies of the diploid human fungal pathogen *Candida albicans*. Eukaryot Cell 4:298–309.

3. Veri AO, Miao Z, Shapiro RS, Tebbji F, O’Meara TR, Kim SH, Colazo J, Tan K, Vyas VK, Whiteway M, Robbins N, Wong KH, Cowen LE. 2018. Tuning Hsf1 levels drives distinct fungal morphogenetic programs with depletion impairing Hsp90 function and overexpression expanding the target space. PLOS Genet 14:e1007270.

4. White TC. 1997. Increased mRNA levels of *ERG16*, *CDR*, and *MDR1* correlate, with increases in azole resistance in *Candida albicans* isolates from a patient infected with human immunodeficiency virus. Antimicrob Agents Chemother 41:1482–1487.

5. Staib P, Morschhäuser J. 1999. Chlamydospore formation on staib agar as a species-specific characteristic of *Candida dubliniensis*. Mycoses 42:521–524.

6. Lockhart SR, Etienne KA, Vallabhaneni S, Farooqi J, Chowdhary A, Govender NP, Colombo AL, Calvo B, Cuomo CA, Desjardins CA, Berkow EL, Castanheira M, Magobo RE, Jabeen K, Asghar RJ, Meis JF, Jackson B, Chiller T, Litvintseva AP. 2017. Simultaneous emergence of multidrug-resistant *Candida auris* on 3 continents confirmed by whole-genome sequencing and epidemiological analyses. Clin Infect Dis 64:134–140.

7. Vyas VK, Barrasa MI, Fink GR. 2015. A *Candida albicans* CRISPR system permits genetic engineering of essential genes and gene families. Sci Adv 1:e1500248.

8. Basso LR, Bartiss A, Mao Y, Gast CE, Coelho PSR, Snyder M, Wong B. 2010. Transformation of *Candida albicans* with a synthetic hygromycin B resistance gene. Yeast 27:1039–1048.
